# Editorial: The immunosuppressive tumor microenvironment and strategies to revert its immune regulatory milieu for cancer immunotherapy

**DOI:** 10.3389/fimmu.2023.1238698

**Published:** 2023-06-26

**Authors:** Mazdak Ganjalikhani Hakemi, Gülderen Yanikkaya Demirel, Yangqiu Li, Nair Jayakumar

**Affiliations:** ^1^ Department of Immunology, Faculty of Medicine, Isfahan University of Medical Sciences, Isfahan, Iran; ^2^ Department of Immunology, Faculty of Medicine, Yeditepe University, Istanbul, Türkiye; ^3^ Institute of Hematology, School of Medicine, Jinan University, Guangzhou, China; ^4^ Women’s Malignancies Branch, National Institutes of Health (NIH), Bethesda, MD, United States

**Keywords:** cancer immunology, cancer immunotherapy, immunosuppression, immune regulation, tumor microenvironment

Despite advancements in tumor immunotherapy, inconsistent therapeutic effects and barriers impacting clinical outcomes highlight the need for a better understanding of the tumor microenvironment (TME) in cancer immunology (1). The TME plays a crucial role in anti-cancer immunity, influencing the effectiveness of immunotherapy and other treatments (2, 3). Interactions between cancer cells, the extracellular matrix, and stromal cells shape the TME, creating a heterogeneous environment that fosters chronic inflammation, immune suppression, and angiogenesis (4–6).

Limited understanding of immune suppression in cancer patients has hindered the success of immunotherapeutic strategies. Therefore, comprehending the TME, tumor immune evasion mechanisms, and the interplay between stromal and immune cells is vital for successful tumor immunotherapy (7, 8). Overcoming immune-suppressive networks and activation barriers within the TME is crucial for effective cancer cell eradication (9–11). Targeting key factors and reprogramming the TME to enhance T cell activity while reducing immune-suppressive cell accumulation are potential strategies.Further studies on TME composition and its impact on immune surveillance attenuation can guide the development of strategies to manipulate the TME and benefit cancer patients (12, 13).

Understanding the TME status, immune cell involvement, and key transcription factors is essential for developing therapies that target inefficient T-cells within the TME. In their study titled “*Regulatory effects of IRF4 on immune cells in the tumor microenvironment*,” Lu et al. demonstrated the significant potential of targeting IRF4 and its interactions with BATF, TCF1, Roquin, or Regnase1 to regulate anti-tumor T-cell immunity and improve therapeutic efficacy. Polyamine metabolism is closely associated with tumor development and the TME. Wang et al. conducted a study on the “*development and validation of polyamine metabolism-associated gene signatures*” to predict prognosis and immunotherapy response in lung adenocarcinoma (LUAD) using machine learning. They identified specific genes related to polyamine metabolism that can predict patient survival and showed their association with immune cell infiltration and immunotherapy response in LUAD patients. Additionally, the role of adenosine triphosphate (ATP) in cellular energy metabolism and the contribution of CD39 and CD73 ectonucleotidases to inflammation, hypoxia, and cancer progression have been recognized as promising therapeutic targets (14, 15). Jiang et al. in a study entitled “*The ectonucleotidases CD39 and CD73 on T cells: The new pillar of hematological malignancy*” highlighted the potential of CD39 and CD73 as disease markers and prognostic indicators in hematological malignancies, contributing to the progression and expansion of leukemias.

Zhou et al. conducted a bibliometric and visual analysis on tumor-associated macrophage (TAM) research, evaluating its research status, focus areas, and development trends. The study covered 6,405 articles published between 2001 and 2021, primarily from the USA and China, providing valuable information for researchers in this promising field of cancer immunology. In their contribution, Zhu et al. explored the inhibition of immune response by stress hormones and its reversal through enhancing the anti-cancer functions of granulocytes using Ginsenoside Rg1, a traditional herbal medicine ingredient. They confirmed the immunoprotective effects of Ginsenoside Rg1 on granulocytes through cell culture and animal experiments. The study demonstrated the downregulation of ARG2, MMP1, S100A4, and RAPSN mRNA expression, as well as the upregulation of LAMC2, DSC2, KRT6A, and FOSB mRNA expression in noradrenaline-inhibited granulocytes. These findings suggest the potential use of Ginsenoside Rg1 as an adjuvant drug for cancer patients experiencing mental stress. Sarsembayeva et al. investigated the role of tumor microenvironment-derived Cannabinoid receptors (CB1 and CB2 receptors) in non-small cell lung cancer. They identified immune cells expressing cannabinoid receptors in the tumor microenvironment and observed that the absence of cannabinoid receptor 2 led to a favorable change in the composition of immune cell populations, favoring tumor-killing lymphocytes. The study indicated that the absence of this receptor significantly improved the response to immunotherapy, highlighting the relevance of microenvironment findings in immunotherapeutic approaches. In the context of immune suppression in solid tumors such as Glioblastoma (GBM), Ni et al. conducted a study titled “*Transcriptome and single-cell analysis reveal the contribution of immunosuppressive microenvironment for promoting glioblastoma progression*.” This research identified immune suppressive subgroups, major cell types, signaling pathways, and molecules involved in the formation of the immune suppressive subgroup. The findings provide valuable insights for future personalized immunotherapy approaches targeting GBM.

In their review titled “*Targeting the Bone Marrow Microenvironment in Acute Myeloid Leukemia: Potential Use of Immune Checkpoint Inhibitors*,” Aru et al. emphasized the impact of dual inhibition of the CXCL12-CXCR4 and PD-1-PD-L1 axes in alleviating the immunosuppressive tumor microenvironment of acute myeloid leukemia (AML). This highlights the potential of immune checkpoint blockade (ICB) as a therapeutic approach for modifying the bone marrow microenvironment (BMM) in AML. However, further research involving larger patient cohorts is needed to fully understand the integration of ICIs in hematological malignancy treatments. Yao et al. explored the immune characteristics of T-cell subsets in peripheral blood and bone marrow samples of chronic myeloid leukemia (CML) patients. They observed altered immune patterns, including increased levels of TIGIT and CD8+ tissue-residual T cells (TRM) in *de novo*-CML patients, while the level of CD8+TEMRA cells decreased in patients who did not achieve a molecular response. These findings suggest that tyrosine kinase inhibitor (TKI) therapy can reshape the T-cell repertoire when patients achieve a molecular response in CML. Lv et al. utilized single-cell RNA sequencing (scRNA-seq) to analyze immune cell dynamics and tumor cell infiltration in the bone marrow (BM) of multiple myeloma (MM) patients. They discovered aberrant metabolic processes associated with the immunosuppressive microenvironment in MM, particularly dysregulated amino acid metabolism that impaired the function of cytotoxic CD8 T cells. The authors propose that restoring metabolic balance should be a key focus for improving the efficacy of immune-based therapies in MM. In the context of B cell malignancies, including MM, B-cell lymphomas, and chronic lymphocytic leukemia, immunomodulatory drugs (IMiDs) such as thalidomide, lenalidomide, and pomalidomide have been employed. Guo et al. summarized the current advances in the use of IMiDs in regulating immune cell function and enhancing the efficacy of immunotherapies across different types of B-cell neoplasms. The authors highlight the importance of IMiDs-based tumor microenvironment re-education as a crucial mechanism for improving treatment outcomes. These studies collectively demonstrate the significance of understanding and targeting the immune disorder within the microenvironment of hematological malignancies, including AML, CML, MM, and B-cell neoplasms. By manipulating the tumor microenvironment, such as through immune checkpoint inhibitors or metabolic interventions, there is potential to enhance the effectiveness of immunotherapies in these diseases.

More recently, an old foe has come back to the forefront of the fight against cancer, namely oncolytic viruses and their more interesting cousins the arenaviruses. While oncolytic viruses have limited efficacy in tumors with intact IFN pathways, arenaviruses provide a promising alternative due to their ability to evade host immunity (16, 17). In their review titled “*Arenaviruses: Old viruses present new solutions for cancer therapy*,” Stachura et al. discuss the resurgence of oncolytic viruses and the emerging use of arenaviruses in cancer treatment. The authors provide a comprehensive overview of arenaviruses, focusing on lymphocytic choriomeningitis virus (LCMV), a non-cytopathic virus with specific cancer tropism. They highlight the recent positive results from early clinical trials with arenavirus-based therapies, presented at the AACR and ASCO meetings in 2023. The review delves into the biology of LCMV, its safety profile in patients, and various LCMV-based therapies and anti-cancer vaccines. The information presented in the review will be valuable for researchers in the field of cancer immunotherapy, providing insights into the potential of arenaviruses as a novel viral-based therapy.

At the whole, we received and enthusiastically reviewed several interesting reviews and research articles on this Research Topic, which shed light on new research directions related to one of the most important and multidisciplinary research subject: “*The Immunosuppressive Tumor Microenvironment and Strategies to Revert its Immune Regulatory Milieu for Cancer Immunotherapy.* “We hope that all the efforts of the editorial team and the articles presented in this Research Topic can be interesting, informative, and inspiring for all our readers, encouraging them to thoroughly explore the presented subject in this Research Topic.

## Author contributions

All authors listed have made a substantial, direct, and intellectual contribution to the work and approved it for publication.
